# Rat embryonic fibroblasts immortalized by MRPS18-2 protein are target for NK-cells

**DOI:** 10.18632/oncotarget.17610

**Published:** 2017-05-04

**Authors:** Muhammad Mushtaq, Pradeepa N. Pangigadde, Suhas Darekar, Erik Dissen, Elena Kashuba

**Affiliations:** ^1^ Department of Microbiology, Tumor and Cell Biology, Karolinska Institutet, Stockholm, Sweden; ^2^ University “Magna Græcia” of Catanzaro, Viale Europa, Catanzaro, Italy; ^3^ Department of Anatomy, Institute of Basic Medical Sciences, University of Oslo, Oslo, Norway; ^4^ R.E. Kavetsky Institute of Experimental Pathology, Oncology and Radiobiology, NASU, Ukraine

**Keywords:** mitochondrial ribosomal protein MRPS18-2, cell immortalization, stem cells, NK killing, cytokine expression profile

## Abstract

Overexpression of the human mitochondrial ribosomal protein MRPS18-2 (S18-2) led to immortalization of primary rat embryonic fibroblasts (REFs). The derived cells (18IM) expressed embryonic stem cell markers. Noteworthy, genes encoding the COX family proteins were up-regulated significantly. It is known that the COX family proteins are involved in the regulation of immune response.

In the present work we demonstrate that 18IM cells behave like stem cells when subjected to directed differentiation *in vitro*. However, unlike stem cells, 18IM cells do not develop tumors *in vivo*, in SCID mice. This phenomenon is observed due to the strong natural killer (NK) cell immunogenicity. 18IM cells were better recognized by NK cells, compared with primary REFs, as was shown by a standard NK killing assay.

Our data explain asymmetry in behavior of stem-like cells *in vivo* and *in vitro*, and this support the notion that stem and/or cancer-initiating cells are preferred targets for NK-cells. Concluding, the S18-2 protein is a putative target for cancer vaccines.

## INTRODUCTION

The S18 family of mitochondrial ribosomal proteins (MRPS18, S18 in the text) consists of three proteins, S18-1-3 [1]. S18 proteins show low sequence homology to each other (about 20 percent), suggesting various functions, as uncharacterized yet.

We have found that overexpression of the S18-2 protein (NP_054765) resulted in immortalization of primary rat embryonic fibroblasts (REFs), associated with induction of stem cell marker expression [2]. In the established cell line (18IM) genes, encoding proteins of the cyclooxygenase (COX) and NADH ubiquinone oxidoreductase (NDUF) families, were significantly up-regulated. It is known that COX and NDUF family proteins are involved in the control on cell proliferation, oxidative phosphorylation, cellular respiration, and other redox reactions. This indicates that 18IM cells are more active metabolically than REFs. The cellular pathways characteristic for rapidly proliferating cells were also activated in 18IM cells, namely the ubiquinone (Coenzyme Q10) biosynthesis, fatty acid elongation in mitochondria, and PI3K/AKT signaling [3]. When grown at a high confluence, 18IM cells demonstrated characteristics of stem cells by differentiating into fat cells spontaneously. Moreover, a proportion of the cells started to express the beta-III-tubulin, MHC class II, and pan-keratin [2].

The 18IM line was not tumorigenic upon inoculation into experimental animals, severe combined immunodeficient (SCID) mice, despite the demonstrated phenotype of the fast proliferating immortalized cells. Actually, small tumors were detected in the first 7-14 days. However, tumors regressed rather fast, in a few weeks in all of animals [3], [4]. Therefore, we asked a question, what are the factors inhibiting growth of 18IM cells *in vivo*? Could the S18-2 protein expressed at the elevated levels evoke immune response?

In the present paper we aimed to explain asymmetry in 18IM growth *in vivo* and *in vitro*.

## RESULTS

### Directed differentiation of 18IM cells

**Figure 1:**
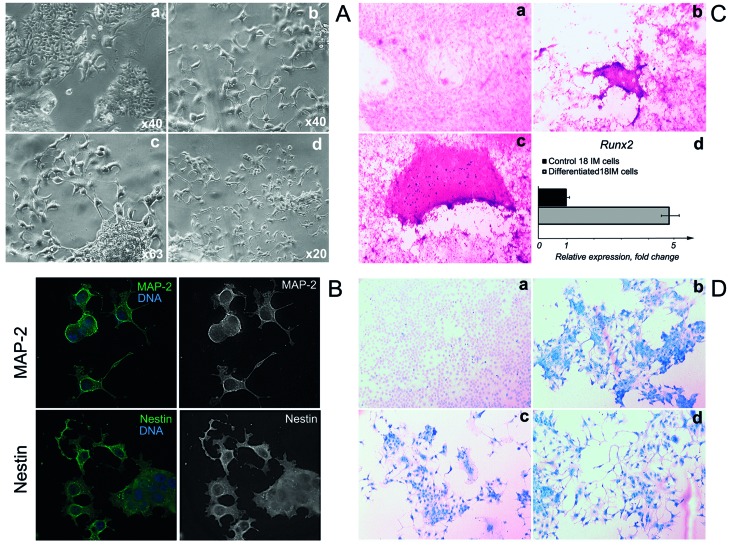
Directed differentiation of 18IM cells *in vitro* **A.** - 18IM cells grown on coverslips were treated for 1 week with 20 µM of RA in culture medium; a - control 18IM cells; b-d, differentiated cells. Notice the change of morphology after treatment with RA. **B.** - 18IM cells were differentiated as in (A). Upper panel - immunostaining with anti-MAP-2 antibody (green); bottom panel - with anti-nestin antibody (green). DNA is shown in blue. Objective was x63. **C.** - 18IM cells were differentiated with a cocktail of dexamethasone, ascorbic acid-2-phosphate, and glycerol-2-phosphate in culture medium for 4 weeks: a - untreated 18IM cells; b-c - differentiated cells, both stained with Alizarin Red S. The strong red signal suggests mineralization of the osteogenic cells. Objective was x40; d - *Runx2* expression at the mRNA level, assessed by q-PCR. **D.** - 18IM cells were differentiated for 4 weeks upon treatment with 0.5 µM and 1 µM of the H-89 in culture medium. The untreated 18IM cells are shown in panel a, and differentiated cells are shown in panels b-d; both are stained with Alcian Blue. Bright blue staining and a change in cell morphology are seen in differentiated cells (panels b-d). Objective was x63.

As was mentioned above, 18IM cells resembled the stem cells and spontaneously differentiated under confluent growth conditions [2]. We aimed to determine whether 18IM cells could be subjected to so-called “directed” differentiation. Accordingly, to promote neurogenic differentiation, 18IM cells were treated with retinoic acid (RA). The first phenotypic changes were observed as early as 48 h after the treatment (Figure 1A). After 1 week, 18IM showed the “glial cell-like” morphology with characteristic filopodia. It is noteworthy that cells proliferated to a certain confluence for approximately 3 weeks, after which a proportion of cells died. A month later, most of the 18IM cells cultured with RA died, as expected for differentiated primary cells. To confirm differentiation into glial cells, after treatment with RA for one week 18IM cells were stained with antibodies against MAP-2 and nestin (Figure 1B). Cells showed strong MAP-2 and nestin signals, suggesting that 18IM cells differentiated into precursors of neuronal cells. These markers showed very weak signal on untreated 18IM cells.

To evoke osteogenic differentiation, cells were cultured in the medium, which contained ascorbic acid-2-phosphate, glycerol, and dexamethasone. Cells grown on ordinary Iscove’s modified Dulbecco’s medium (IMDM) were used as internal controls. To monitor mineralization, cells were stained with a solution of Alizarin Red S. Strong signal was detected on a proportion of 18IM cells after differentiation (Figure 1C, panels b and c), by contrast with the control 18IM cells (Figure 1C, panel a). The dark red signal was somewhat diffuse because of the use of the Alizarin Red solution at the low pH (4.6). Under such conditions, the partial removal of calcification in tissues has been observed [5]. To confirm osteogenic differentiation, expression levels of the *Runx2* (NM_001278483) were assessed by Q-PCR. *Runx2* encodes a transcription factor that is essential for the maturation of osteoblasts and is expressed at higher levels upon osteogenic differentiation [6]. *Runx2 e*xpression was elevated significantly in differentiated 18IM cells (Figure 1C, panel d).

To induce chondrogenesis, 18IM cells were grown in a medium supplemented with H-89. We found that cell morphology was changed significantly after 4 weeks (Figure 1D, b-d). To monitor negatively charged molecules, such as RNA, DNA, glycosaminoglycans, and proteoglycans, cells were stained with Alcian Blue solution. As seen on Figure 1D, 18IM cells were negative for Alcian Blue staining (panel a), while cells showed an intense blue signal after H-89 treatment (Figure 1, panels b-d). Blue dye was detected in the cytoplasm and membranes of the differentiated cells, suggesting the production of acid glycosaminoglycans that are the elements of the extracellular matrix.

Hence, the 18IM cells demonstrated features of stem cells. We wondered whether 18IM cells differs from the primary REFs also by expression profiles of inflammatory cytokines and receptors, that is characteristic for the stem and cancer-initiating cells.

### Expression pattern of inflammatory cytokines and receptors

**Figure 2:**
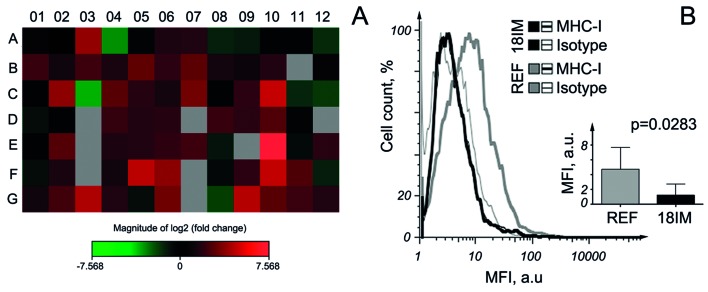
Expression of chemokines and their receptors in 18IM cells, compared with REFs **A.** - The heat map of the fold changes in gene expression between 18IM cells and REFs for every gene in the array, in the context of the array layout (see Figure S1 for the genes). **B.** - 18IM cells and primary REFs were incubated with either anti-MHC-I or unrelated isotype control antibodies. A representative FACS analysis showing the difference in MHC-I molecule expression between REFs and 18IM cells; the inserted panel shows statistical analysis of MHC-I expression from three independent experiments, using two tailed paired T test on the combined mean values. MFI - the mean fluorescence intensity; a. u. - arbitrary units.

To analyze the expression of genes related to the different inflammatory pathways at the mRNA level, the RT^2^ profiler assay was performed, using RNA isolated from 18IM cells and REFs for comparison. After an analysis of the quantitative PCR (q-PCR) data, genes that showed at least 4 folds difference in expression levels between 18IM and REFs were selected. Genes on the plate represented mainly chemokines, cytokines and their receptors. 26 genes out of 84 analyzed were expressed in 18IM at the higher levels compared with REF (Table 1, Figure 2A, and Supplementary Table 1). All of these genes encode proteins that induce pro-inflammatory effects.

**Table 1:** The list of cytokines, chemokines and their receptors that were expressed at the mRNA levels at ≥4 fold in 18IM cells, compared with REFs.

Genes upregulated in 18IM cells compared with REFs			
Cytokine/Receptor	Accession number	Binding receptor/cytokine	Relative fold change
*Ccl11*	NM_019205	CCR2, CCR3, CCR5	15.73
Ccl5	NM_031116	CCR1, CCR3, CCR5	4.58
*Ccl7*	NM_001007612	CCR1, CCR2, CCR3	4.94
*Ccr1*	NM_020542	CCL3, CCL5, CCL7, CCL23	7.92
*Ccr2*	NM_021866	CCL1	7.59
*Csf1*	XM_008761426	CSF1R (CD115)	14.57
*Csf3*	NM_017104	CSF3R	7.20
*Cxcl1*	NM_030845	CXCR2 (IL8RB, IL8R2)	9.99
*Cxcl12*	NM_022177	CXCR4, CXCR7	28.78
*Cxcr5*	NM_053303	CXCL13	4.47
*Il11*	NM_133519	IL11RA	4.49
*Il15*	NM_013129	IL15A	6.31
*Il17f*	NM_001015011	IL17RA?	6.61
*Il1rn*	NM_022194	IL1R1	4.10
*Il21*	NM_001108943	IL21R	4.91
*Il2rg*	NM_080889	IL2RA, IL4RA, IL7RA, IL9RA, IL15RA, IL21RA	189.76
*Il6st*	NM_001008725	IL6:IL6RA, IL6/IL12 complexes	25.97
*Il7*	NM_013110	IL7R, TCRß, HGF	13.92
*Tnfsf10 (TRAIL)*	NM_145681	TRAILR1 (DR4), TRAILR2 (DR5), TRAILR3, TRAILR4	9.72
*Tnfsf13b*	NM_001109112	TNFRSF13B (TACI), TNFRSF17 (BCMA), TNFRSF13C (BAFF-R)	23.46
*Tnfsf14*	NM_001191803	TNFRSF14	7.29

Gene name	Accession number	Description	Relative fold change
*Mif*	NM_031051	MIF, MHC class II	27.82
*Spp1*	NM_012881	This gene encodes osteopontin, or secreted phosphoprotein	21.81
*Tnfsf4*	NM_053552	This gene encodes glycoprotein Gp34	4.77

Genes downregulated in REFs compared with 18IM			
Cytokine/Receptor	Accession number	Binding receptor/cytokine	Relative fold change
*Ccl12*	NM_001105822	CCR2	8.64
*Csf2*	NM_053852	CSF2R (CD116)	11.29

It is well known that the major histocompatibility complex (MHC) class I molecule, one of the most important players involved in immune response, is expressed at lower levels in stem and cancerous cells [7]. MHC class I molecule expression levels were measured, using a specific anti-MHC-I antibody. Indeed, levels of MHC class I protein on 18IM cells were significantly lower than those measured on their REF counterparts (Figure 2B).

The decreased levels of MHC class I molecules on the surface of virus-infected and cancer cells make them targets for NK cells, we wondered whether this also apply for 18IM cells.

### 18IM cells showed higher susceptibility for natural cytotoxicity

To determine whether 18IM cells show susceptibility to recognition and killing by NK cells, an *in vitro* NK cell killing assay for 18IM cells in comparison with primary REFs was performed, using rat splenocytes. REFs described earlier [2] and rat splenocytes, used in this study, were both derived from the Sprague Dawley (SD) rats; hence, 18IM cells, REFs, and splenocytes could be considered as isogenic.

**Figure 3:**
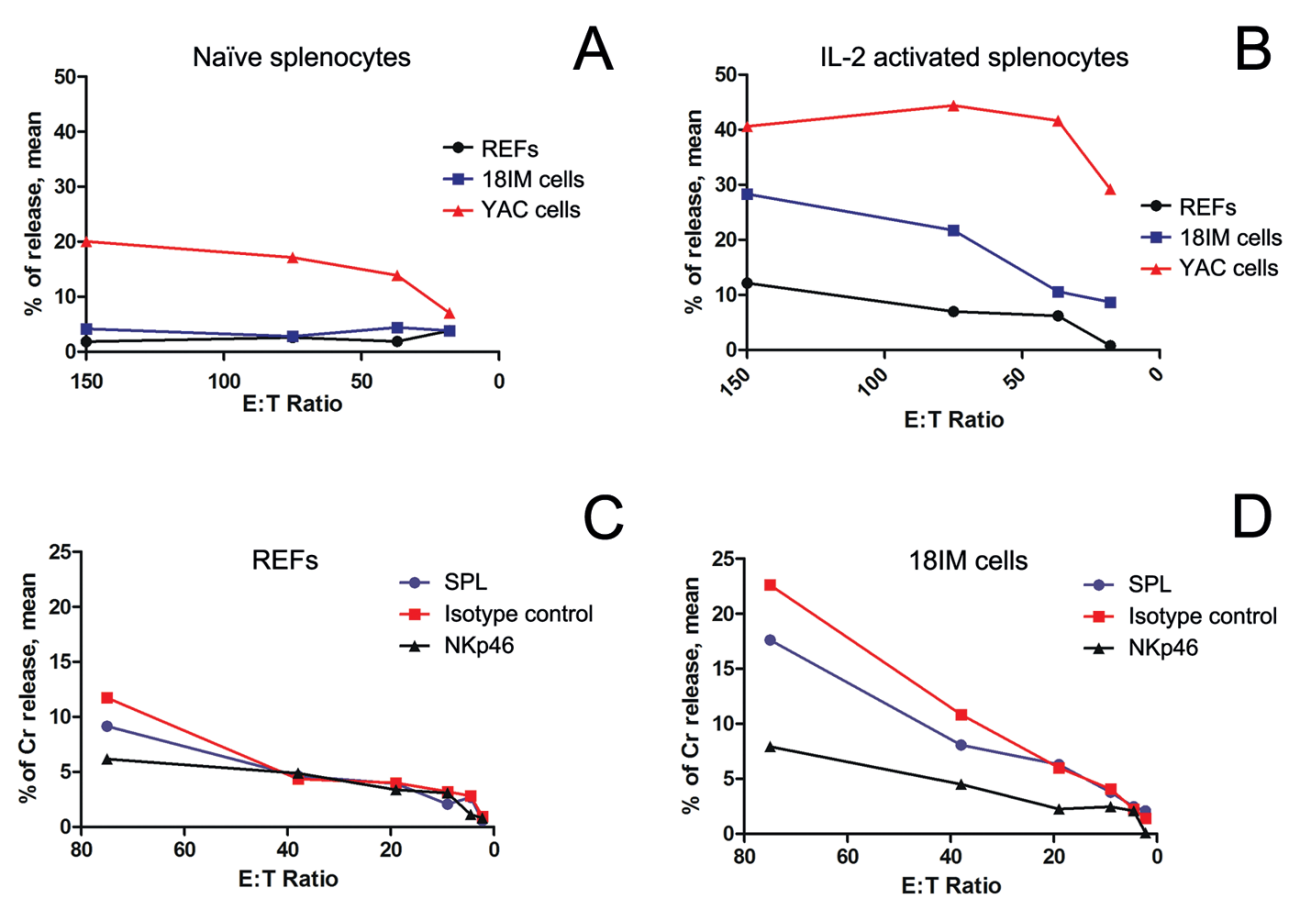
The cytotoxic recognition of REFs and 18IM cells by splenocytes, studied at various splenocyte-to-target-cell (E:T) ratios Rat splenocytes were used as effector (E) cells, and REFs and 18IM cells as targets (T) in the assay. Non-parametric t-test (panels A and B) and Wilcoxon signed rank test (C and D) were used to compare a median of three different experiments, performed in triplicates for all the E:T ratios. **A.** - No differences were observed between REFs and 18IM cells for naïve splenocytes (*p* = 0.0747). YAC - control mouse lymphoma YAC-1 cell line. **B.** - The IL-2-activated rat splenocytes preferentially recognize 18IM cells, compared with primary REFs (*p* = 0.0305). **C.** - NKp46 blocking assay for REF recognition by activated splenocytes: SPL treated with anti-NKp46 antibody (NKp46, *p* = 0.0938), and SPL treated with the isotype control antibody (isotype control, *p* = 0.0625). **D.** - NKp46 blocking assay for 18IM cell recognition by activated splenocytes: SPL treated with anti-NKp46 antibody (NKp46, *p* = 0.0320), and SPL treated with the isotype control antibody (isotype control, *p* = 0.0625).

Initially, REFs and 18IM cells were presented to the naïve NK cells (i.e., not activated). No significant differences were observed in the killing pattern of REFs and 18IM cells (Figure 3A). By contrast, when splenocytes were activated with interleukin 2 (IL-2), their recognition of 18IM cells and REFs was dissimilar (Figure 3B). 18IM cells showed higher susceptibility to NK-recognition, compared with REFs. A cytotoxic effect was observed at even low splenocyte/rat cell (E: T, Effector: Target) ratios, suggesting that the cytotoxic reaction occurs also in anatomical compartment where NK cells are poorly represented.

To determine the mechanism responsible for the observed natural cytotoxic effect, a specific antibody against activating receptor NKp46 (or the control antibody, isotype matched) was added to the lymphocytotoxicity assays. As shown in Figure 3C, no change in REF lysis was observed. On contrary, treatment with anti-NKp46 antibody, but not with the isotype control antibody, prevented the selective killing of 18IM target cells (Figure 3D).

Taking into consideration that 18IM killing was mediated by NK cells, we asked a question whether this process may take place in experimental animals, SCID mice.

### 18IM cells were recognized by NK cells of SCID mice

**Figure 4:**
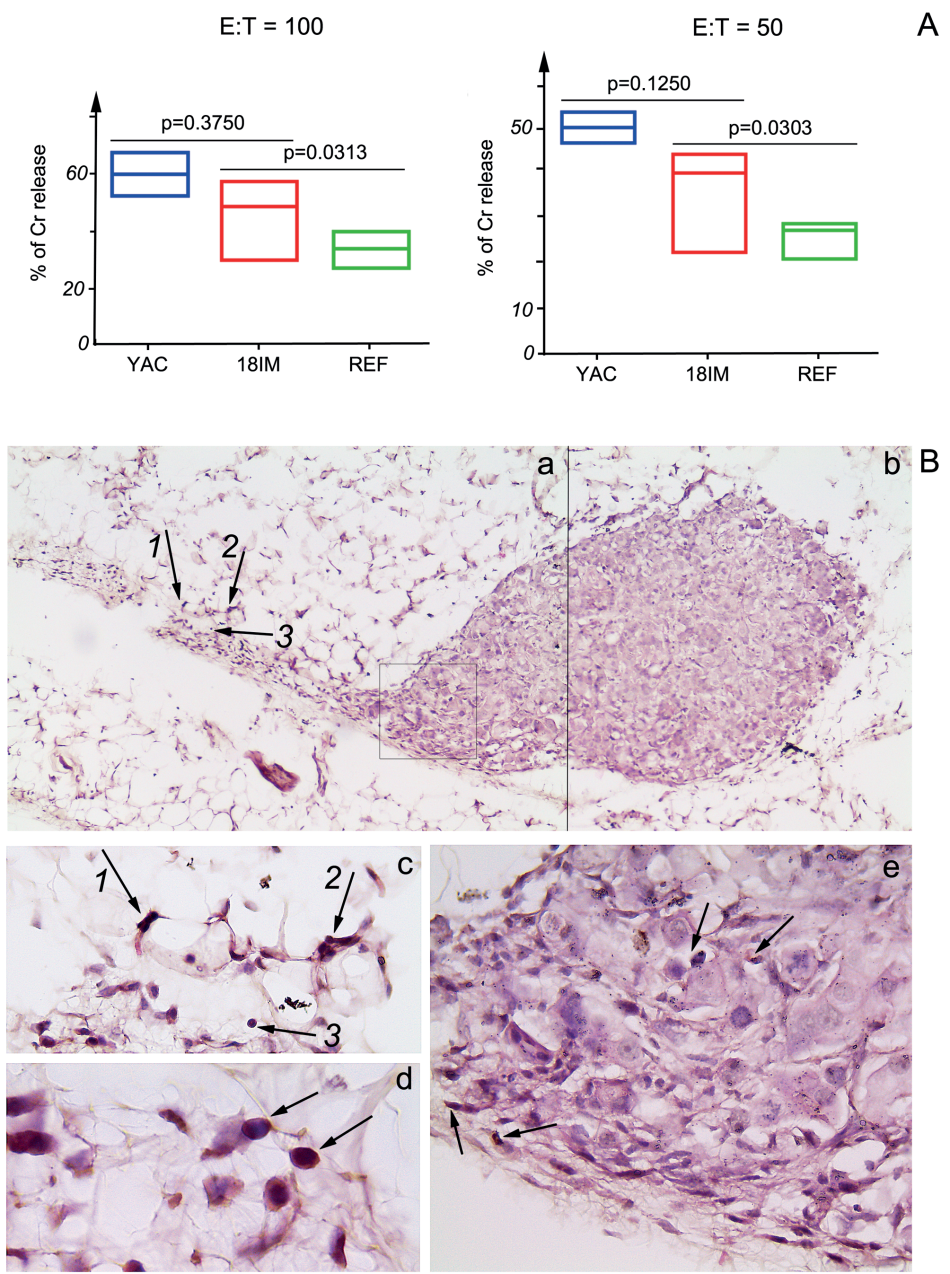
Recognition of 18IM cells by NK-cells of SCID mice *in vitro* and *in vivo* **A.** - Splenocytes of SCID mice were used as effector (E), and 18IM cells and REFs as targets (T) in the *in vitro* NK cell cytotoxicity assay. The cytotoxic recognition was studied at E:T ratios 100:1 and 50:1. Non-parametric t-test was used to compare a median of three different experiments, and for one experiment a pool of the activated NK cells from two spleens was used. 18IM cells were killed by the IL-2 activated NK cells more efficiently than REFs (*p* = 0.0313 for E:T = 100:1 and *p* = 0.0303 for E:T = 50:1), while no significant differences were observed between 18IM and the control mouse lymphoma YAC-1 (YAC) cells killing. **B.** - Tissue sections show a bulk of 18IM cells and NKp46-positive cells in the proximity of the former (black arrows on the panels a, c, d, and e). Panels a and b are merged from the two sequentially captured images with objective x10. Black arrows with numbers on the panel a correspond to those on the panel c (the image on c was captured with objective x40). Notice the cytoplasmic NKp46 signal (brown) on the panel d (objective was x100). 18IM cells that show S18-2 signal (pink) on the panel e were captured with objective x40 (the image corresponds to cells framed in the square on the panel a).

To find out whether 18IM cells could be recognized and killed by NK cells of experimental animals (SCID mice), *in vitro* NK cell killing assay was performed, using splenocytes isolated from SCID mice. Three experiments for 18IM cells and REFs were performed, and for one experiment a pool of the activated NK cells from two spleens were used. NK cells of SCID mice killed 18IM successfully, in comparison with REFs (Figure 4A).

The next question was whether NK cells could be detected in proximity to 18IM cells in SCID mice. Therefore, 18IM cells were inoculated into SCID mice subcutaneously and after 1 week the tissue was isolated. Tissue sections were stained with antibodies against S18-2 and NKp46. The strong signal of NKp46 was detected on the membrane of small cells (Figure 4B, brown) at the inoculation site.

Next, we have diminished expression of the S18-2 in 18IM cells, using a cocktail of siRNAs for 24 h (see Supplementary Figure S1 online). We have to mention, that 18IM cells were treated with the mixture of siRNAs only for 24 h, because the longer treatment (≥ 48 h) induced the cell death, as we have shown earlier [2]. The treated cells were introduced into SCID mice subcutaneously, and the animals were observed for 6 weeks. In contrast to the established 18IM cells, the formation of small tumors was detected in all 4 spots (in two mice), along with inflammation (see Supplementary Figure S2 online, left and right panels, respectively).

## DISCUSSION

NK-cells, a subset of innate lymphoid cells (ILCs), express both, inhibitory and activating receptors on their surface. The inhibitory receptors (KIRs in human, Ly49 in rodents) recognize MHC class I or MHC class I-like molecules. Activating receptor NKG2D (KLRK1, NP_031386) binds to stress-inducible activating ligands, such as MIC-A, MIC-B, and ULBPs in humans and RAE, H60, and MULT1 in mice, respectively [8]. However, the NKp46 ligands (human NCR1, NP_001138929) are still poorly characterized in both species. When the equilibrium between inhibitory and activating receptors is shifted, downregulation of MHC class I expression or the presence of activating ligands induces NK-cells to kill the targets. Malignant transformation and viral infection result in decreased expression of MHC class I molecules, as a rule. Rapid killing of tumor cells that were deficient in MHC class I molecules was observed both *in vivo* and *in vitro* [9], [10], [11]. Noteworthy, NK cells preferentially target human cancer-initiating cells *in vitro* [12], [13].

Stem cells, especially embryonic stem cells (ESC) form teratomas in experimental animals and tumor growth depends on the immune response of the host. It was shown, that NK-cells could inhibit (partially) growth of tumors, formed by mouse ESCs. The tumor regression was observed in one out of 14 animals upon inoculation of 1∙10^6^ of ESCs into SCID mice [14]. Moreover, rat NK cells (isolated from spleen) killed efficiently ESCs in contrast to their differentiated counterparts, as was shown by the chromium release assay *in vitro* [14]. Noteworthy, the MHC class I was expressed at very low levels on the surface of ESCs, while a reciprocal expression pattern was observed for the ligands for the activating receptor NKG2D [7]. Similar phenomenon was observed with human induced pluripotent stem cells (hiPSCs): IL-2 activated NK-cells recognized and killed the former, and this event was partially regulated by the activated NK-cell receptor DNAM-1 (CD226, NP_001290547). Moreover, hiPSCs expressed CD112 (PVRL2, NP_001036189) and CD155 (PVR, NP_001129240) proteins that are ligands for DNAM-1 [15]. Interaction between NK-cell activating receptor NKG2D and its ligands, MICA (NP_000238) and MICB (NP_001276089) played an important role in eradication of hiPSCs.

Our results show that the 18IM cells, resembling ESCs, were not tumorigenic in experimental animals, SCID mice, due to their NK susceptibility and probable trans-differentiation.

It is known that chemokines regulate various biological functions of NK cells. Like IL2, CC chemokines can induce the proliferation and activation of killer cells (see ref. [16]). Chemokines activate NK cells to become the highly cytolytic cells, known as the CC chemokine-activated killer (CHAK) cells that are able to kill tumor cells [17]. It was shown that certain chemokines are important for the CHAK activity of NK cells [18]. The CCL5, CCL7, CCL11, CXCL1, CXCL12, CCR1, and CCR2 were listed as activating chemokines or their receptors [19].

Noteworthy, we found that these molecules were also expressed in 18IM at the significantly higher levels, compared to REFs. This may add further evidences that can explain why 18IM cells were preferentially targeted by NK cells, compared with REFs.

Importantly, NK cells were detected at the inoculation site after 7 days of grafting, as was shown by immunohistochemistry. We may speculate that the chemokine and chemokine receptors expressed at the surface of 18IM cells induced homing of NK cells to the introduced cells. Thus, it was shown before, that NK cells were recruited towards CCL2 and CCL5 [20], and CXCL10, together with CXCL11 directed NK cells to the MHC class I deficient cells [21]. CX3CL1 and CX3CR1 that were also upregulated in 18IM cells were shown to be involved in eradication of metastasis [22]. When the levels of S18-2 protein were diminished (after the treatment with the specific siRNAs), the small tumors were formed. Hence, we may conclude on that non-tumorigenic behavior of 18IM cells in SCID mice is due to activity of NK cells, activated by chemokines and their receptors expressed by 18IM cells.

**Figure 5:**
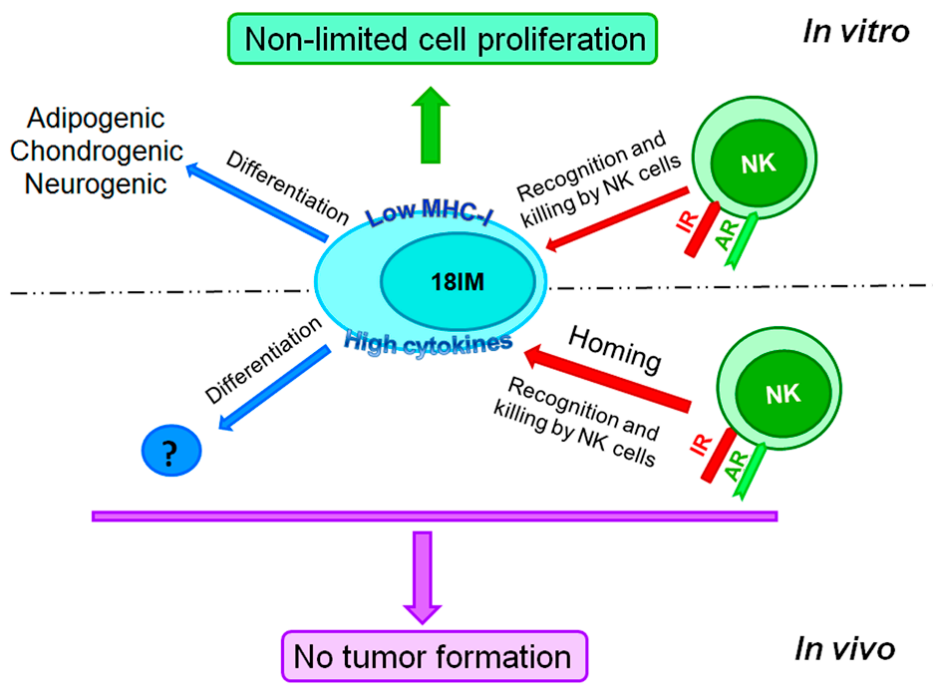
18IM cells are immortalized but do not produce tumors in experimental animals, showing the asymmetric behavior *in vitro* and *in vivo* 18IM cells are recognized (and killed, eventually) by NK cells of SCID mice. They can be trans-differentiated after inoculation as well. AR - activating receptors on NK cells; IR - inhibitory receptors on NK cells.

Beside activation of NK cells, 18IM cells can also be differentiated by treatment with cocktails of various chemicals, not only spontaneously, as we found earlier [2]. Moreover, the decreased expression of surface MHC class I, the main inhibitory molecule for NK cells recognition was shown for the 18IM cells (summarized on Figure 5).

Our data allows us speculate that the S18-2 protein plays an important role in carcinogenesis and modulation of the cell immunogenicity. The molecular mechanisms of cell immortalization upon overexpression of S18-2 and the role of S18-2 in cell cycle control and carcinogenesis are under current investigation.

The susceptibility of S18-2-transfected cells to NK cells suggests that NK cells may be involved in immune intervention in cancers overexpressing S18-2. These new findings will open new avenues of research and have important implications for both basic and clinical science.

## MATERIALS AND METHODS

### Cell culture and immunostaining

Primary REFs and 18IM cells (i.e., REFs, immortalized by the overexpression of S18-2 [2]) were used in the present study. All cells (except for those used in differentiation studies) were cultured at 37°C in Iscove’s medium that contained 10% fetal bovine serum and appropriate antibiotics.

To diminish the S18-2 levels, a mixture of the four siRNAs was used (Thermo Scientific), according to the manufacturer’s protocol, as described in details earlier [2]. Each of 4 siRNA could target the coding region of *S18-2*. The cell lysates were prepared, using NP40-containing buffer (see [2] for details). After western blotting (using 12% SDS-polyacrylamide gel), membranes were stained with rabbit polyclonal antibodies against anti-S18-2 (Proteintech Group, Inc.) and GFP (Cell Signaling Technology), and also with mouse monoclonal anti-actin antibody (Sigma-Aldrich). To visualize protein bands, the ECL kit, anti-mouse and anti-rabbit horseradish peroxidase conjugated secondary antibodies (produced in sheep and donkey, correspondingly) were used (GE Healthcare Bio-Sciences AB).

Before staining, cells were grown on coverslips in six-well plates and fixed in a mixture of methanol and acetone (1:1) at -20°C. Cells were rehydrated in phosphate-buffered saline (PBS) and stained. The following primary antibodies were used: anti-nestin and anti-MAP-2 (Cell Signaling Technology), and conjugated with fluorochrome PE the OX-18 (Novus Biologicals). FITC-conjugated rabbit anti-mouse serum (Dako) was used as a secondary antibody. Hoechst 33258 (Sigma-Aldrich) was added at a concentration of 0.4 µg/mL in the secondary antibody solution for DNA staining. Images were captured using a Leitz DM RB DAS microscope fitted with a dual-mode cooled charge-coupled device (CCD) camera (C4880, Hamamatsu).

### Directed differentiation

To evoke the neurogenic differentiation, 18IM cells were treated with RA solutions in DMSO, and the final concentration of RA was 10 µM and 20 µM, correspondingly.

To perform osteogenic differentiation, 18IM cells and the control REFs were grown in IMDM medium, supplemented with dexamethasone (0.1 µM), ascorbic acid-2-phosphate (0.2 mM), and glycerol-2-phosphate (10 mM), as described previously [23]. Growth medium was changed three times per week. After 2 and 4 weeks, cells were stained with a 2% (w/v) solution of Alizarin Red S (Sigma-Aldrich), which forms complexes with the calcium ions produced by osteocytes [24]. The pH of the solution was adjusted to 4.6, using 10% ammonium hydroxide. Cells were incubated for 30 min in the Alizarin Red S solution and washed six times with water. To monitor chondrogenic differentiation, cells were grown in Glasgow minimum essential medium (G-MEM) supplemented with nonessential amino acids, sodium pyruvate, and 0.1 mM beta-mercaptoethanol, according to the method described previously [4]. The N-(2-(4-bromocinnamylamino)ethyl)-5-isoquinolinesulfonamide (H-89), an inhibitor of a protein kinase A, was used previously to induce chondrogenesis of rat mesenchymal stem cells at 0.1-1.0 µM [25]. In our experiments, the medium in two wells contained 0.5 µM of H-89 (Adipogen International), and in two other wells, 1 µM of H-89. Cells in the remaining two wells were used as negative controls. After 4 weeks, cells were stained with Alcian Blue (Sigma-Aldrich). Cells were incubated in a solution of 1% (w/v) Alcian Blue in 3% acetic acid (the pH of the staining solution was 2.5) for 30 min as previously described [26]. Cells were then washed six times with water.

### Quantitative PCR (q-PCR)

Total RNA was isolated from cells before and after osteogenic differentiation, using an RNeasy Mini kit (Qiagen Inc). Approximately 1 µg of total RNA was used for cDNA synthesis, using a First Strand Synthesis Kit (Sigma-Aldrich), according to the manufacturer’s protocol. Primer concentration was adjusted to a final concentration of 3 µM. Total reaction volume for all q-PCR experiments was 20 µl. Q-PCR was performed, using an SYBR Green Master Mix on a 7900 machine (Applied Biosystems, Foster City, CA, USA). The following primers for the rat *Runx2* were used: Runx2_For 5’-ACACCGTGTCAGCAAAGC-3’; Runx2_Rev 5’-GCTCACGTCGCTCATCTTG-3’. As an internal control for standardizing expression, a gene encoding TATA-Binding protein (*Tbp*, NM_001004198) was assayed. The following primers were used: For 5’-TTTCTTGCCAGTCTGGAC-3’, Rev 5’-CACGAACCACGGCACTGATT -3’. The PCR cycling conditions were: 10 min at 95°C, 40 cycles of 10 s at 95°C, and 1 min at 60°C. Applied Biosystems 7900 system software was used for analysis. Ct values were determined for the internal control (*Tbp*) and *Runx2* at the same threshold in the exponential phase of PCR curves. Relative quantification (comparative Ct (∆∆Ct) method) was used to compare the expression levels. Dissociation curve analysis was performed after every run to check the specificity of the reaction. For each analysis, 3-5 reactions (each in triplicate) were run, and a standard deviation was calculated.

### Study on expression pattern of inflammatory cytokines and receptors by RT2 profiler assay

Cell pellets were re-suspended in TRIzol Reagent (Sigma-Aldrich). Using RNeasy Mini kit (Qiagen) total RNA was isolated from cells, following the manufacturer’s instructions. 2µg of total RNA was used to prepare cDNA using RT^2^ first strand kit (Qiagen). The RT^2^ Profiler PCR Array for rat inflammatory cytokines and receptors (SA Bioscience, Frederick, MD, USA) was used to determine the expression pattern of 84 genes involved in immune response. The q-PCR was performed, using 2µg of cDNA and Real Time PCR System 7300 (Applied Biosystems). The obtained CT values were uploaded on the manufacturer website for online analysis of gene expression.

### Splenocyte preparation

Permission to perform specified procedures on rats and SCID mice was obtained by the Solna court decision (number 290/11 from August, 31th, 2011 and 192/14 from October 9th, 2014, respectively).

Spleens were harvested from the SD rats (Scanbur AB). They were placed in a petri dish and cut into 2-3 pieces in 4 ml of IMDM medium. Collagenase (400 U/ml) was added, and the mixture was incubated at 37°C for 25 min. EDTA was immediately added at a final concentration of 2 µM, and the mixture was incubated for 5 min more. The spleens were mashed, and the suspension was collected in a 15 ml Falcon tube to wash the cells. Subsequently, 10 ml of a lysis buffer (BD Biosciences) was incubated with the pellet at room temperature for 2 min. After washing, the pellet was resuspended in 5 ml of RPMI and passed through a cell strainer to obtain a single cell suspension of splenocytes.

Similarly, splenocytes were isolated from 6 spleens of SCID mice, 2 spleens for each experiment. After 4 days of activation by IL-2, NK cells were separated from spleenocytes using NK Cell Isolation Kit II, mouse (Miltenyi Biotec Inc, USA).

### Cytotoxicity and NKp46 receptor blocking assay

Splenocytes were activated with 1000 U/ml of IL-2 for 42 h (4 days in the case of mouse cells) and were used as effectors in a standard ^52^Cr release assay. As control target cells, the mouse lymphoma cell line YAC-1 was used. To block the NKp46 receptor, effectors were treated with anti-rat mouse monoclonal antibody WEN23 (whole IgG) that was a generous gift from Eric Dissen (Department of Anatomy, Institute of Basic Medical Sciences, University of Oslo, Norway, described in ref. [27]). Mouse IgG2a type KIR2DL2/L3 antibody (BioLegend) was used as an isotype control. In these assays, antibodies were used at the concentration 10 µg/ml.

### Study on 18IM cells tumorigenicity in SCID mice and immunohistochemistry

The 1-2∙10^6^ of 18IM cells were inoculated into SCID mice subcutaneously. After 7 days mice were sacrificed and biopsies were fixed in a neutral buffered 4% formaldehyde solution. After fixation, dehydration, and embedding in paraffin, serial sections were cut at a normal thickness of 5 μm. Paraffin was dissolved in xylol and the tissue was rehydrated by stepwise washing with EtOH in PBS (99%, 90%, 70%, and 30% EtOH). Tissues were then treated in a 2% solution of H_2_O_2_ in methanol at room temperature for 30 min to reduce background staining. Epitopes were exposed by hot citrate buffer (water bath, 92°C for 15 min). Rabbit polyclonal anti-S18-2 (Proteintech Group Inc) and rat anti-mouse NKp46 (CD335) (clone 29A1.4, BioLegend) antibodies were diluted (1:200 and 1:100) in blocking buffer (2% bovine serum albumin, 0.2% Tween-20, 10% glycerol, and 0.05% NaN3 in PBS). The NKp46 and S18-2 signals were visualized with the help of the EnVision™ Detection systems (Dako) by Peroxidase/DAB and Permanent Red, respectively. Nuclei were stained with the Mayer’s hematoxylin (Dako).

## SUPPLEMENTARY MATERIALS FIGURES AND TABLE


