# Supplementary material for: Rat embryonic fibroblasts immortalized by MRPS18-2 protein are target for NK-cells
Source: Oncotarget. 2017 May 4;8(39):64907–17. doi: 10.18632/oncotarget.17610 (PMC5630300; doi:10.18632/oncotarget.17610)
Supplement: Supplementary file 1 [file oncotarget-08-64907-s001.pdf]

Rat embryonic fibroblasts immortalized by MRPS18-2 protein are target for NK-cells

Supplementary Material

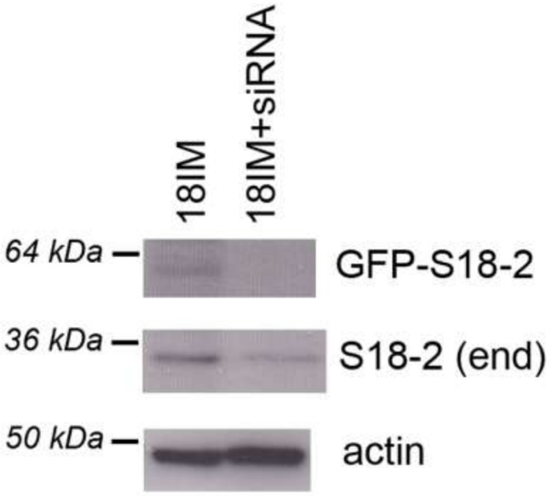

Supplementary Figure S1: The levels of S18-2 protein were diminished upon the treatment of 18IM cells with a cocktail of siRNAs. 18IM cells were treated with a mixture of siRNA targeting *S18-2* cDNA for 24 h. Proteins in the cell lysates were separated, using electrophoresis on 12% polyacrylamide SDS gel. The membrane was probed with the rabbit antibodies against GFP and S18-2, and mouse monoclonal anti-actin antibody.

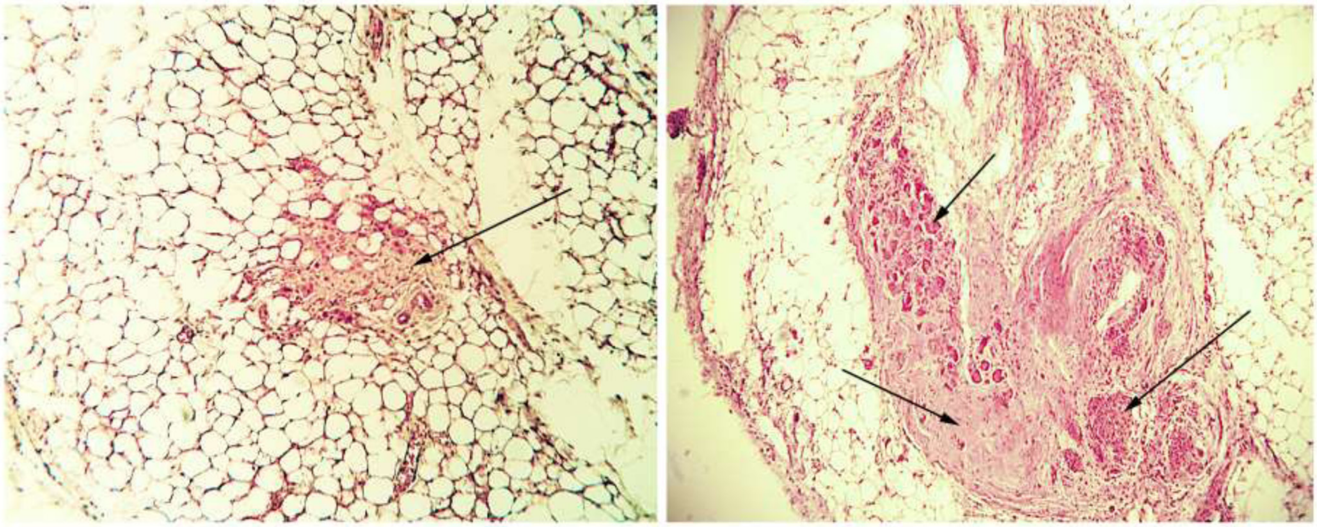

Supplementary Figure S2: 18IM cells with decreased levels of S18-2 formed small tumors in SCID mice. The 18IM cells treated with siRNA for 24 h were introduced into SCID mice subcutaneously (2 mice, 4 spots altogether), and the animals were observed for 6 weeks. Small tumors were detected, along with inflammation

SupplementaryTable S1: The report on a gene expression analysis performed, using the RT^2 profiler PCR array rat inflammatory cytokines and receptors.*

	01	02	03	04	05	06	07	08	09	10	11	12
**A**	Aimp1<br>1.60	Bmp2<br>1.01	**Ccl11**<br>15.73	Ccl12<br>-8.64	Ccl17<br>-1.21	**Ccl19**<br>2.95	**Ccl2**<br>3.12	Ccl20<br>-2.59	Ccl22<br>-2.28	Ccl24<br>1.19	Ccl3<br>-1.13	Ccl4<br>-3.09
**B**	**Ccl5**<br>4.58	Ccl6<br>2.34	**Ccl7**<br>4.94	**Ccl9**<br>2.84	**Ccr1**<br>7.92	Ccr10<br>3.95	**Ccr2**<br>7.59	Ccr3<br>2.6	Ccr4<br>2.11	**Ccr5**<br>3.24	Ccr6<br>-3.84	**Ccr8**<br>1.60
**C**	Cd40lg<br>1.33	Csf1<br>14.57	Csf2<br>-11.29	Csf3<br>7.20	**Cx3cl1**<br>3.64	**Cx3cr1**<br>2.56	Cxcl1<br>9.99	**Cxcl10**<br>1.31	**Cxcl11**<br>3.85	**Cxcl12**<br>28.78	Cxcl2<br>-2.84	Cxcl5<br>-3.63
**D**	Cxcl9<br>-1.79	Cxcr2<br>1.30	Cxcr3<br>-3.84	Cxcr5<br>4.47	Fasl g<br>3.33	Ifng<br>3.62	Il10ra<br>-3.84	Il11<br>4.49	Il13<br>3.79	Il15<br>6.31	Il16<br>-1.28	Il17a<br>-3.84
**E**	Il17b<br>-1.15	Il17f<br>6.61	Il1a<br>-3.84	Il1b<br>3.07	Il1r1<br>3.28	Il1rn<br>4.1	Il21<br>4.91	Il27<br>-2.13	Il2rb<br>-3.84	Il2rg<br>189.76	Il3<br>-1.64	Il33<br>3.72
**F**	Il4<br>-1.79	Il5<br>2.83	Il5ra<br>-3.84	Il6r<br>2.5	Il6st<br>25.97	Il7<br>13.92	Cxcr1<br>-3.84	Lta<br>-1.59	Ltb<br>3.96	Mif<br>27.82	Nampt<br>6.13	Osm<br>-2.45
**G**	Pf4<br>2.35	RGD1561905<br>5.13	Spp1<br>21.81	Tnf<br>3.15	Tnfrsf11b<br>-1.77	Tnfsf10<br>9.72	Tnfsf11<br>-3.84	Tnfsf13<br>-3.81	Tnfsf13b<br>23.46	Tnfsf14<br>7.29	Tnfsf4<br>4.77	Vegfa<br>3.21

*- 96 genes (12 of them – positive and negative controls) were profiled on 2 samples (in duplicates) with the PARN-011Z. Control group –REFs, group 1 – 18IM cells. The C_T cut-off was set to 36. The cytokines marked in red color are involved in the CHAK activity of NK cells.
